# How to Be a Copenhagenistic-QBistic Everettist

**DOI:** 10.3390/e27030248

**Published:** 2025-02-27

**Authors:** Marcin Wieśniak

**Affiliations:** Institute for Theoretical Physics and Astrophysics, Faculty of Mathematics, Physics, and Informatics, University of Gdańsk, 80-308 Gdańsk, Poland; marcin.wiesniak@ug.edu.pl

**Keywords:** quantum measurement, foundations of quantum mechanics, interpretation of quantum mechanics

## Abstract

The measurement problem in quantum mechanics (QM) is related to the inability to include learning about the properties of a quantum system by an agent in the formalism of quantum theory. It includes questions about the physical processes behind the measurement, uniqueness, and randomness of obtained outcomes and an ontic or epistemic role of the state. These issues have triggered various interpretations of quantum theory. They vary from refusing any connection between physical reality and a measurement process to insisting that a collapse of the wave-function is real and possibly involves consciousness. On the other hand, the actual mechanism of a measurement is not extensively discussed in these interpretations. This essay attempts to investigate the quantum measurement problem from the position of the scientific consensus. We begin with a short overview of the development of sensing in living organisms. This is performed for the purpose of stressing the relation between reality and our experience. We then briefly present different approaches to the measurement problem in chosen interpretations. We then state three philosophical assumptions for further consideration and present a decomposition of the measurement act into four stages: transformation, conversion, amplification and broadcasting, and, finally, perception. Each of these stages provides an intuition about the physical processes contributing to it. These conclusions are then used in a discussion about, e.g., objectivity, the implausibility of reversing a measurement, or the epistemic status of the wave-function. Finally, we argue that those in favor of some of the most popular interpretations can find an overlap between their beliefs and the consequences of considerations presented here.

## 1. Introduction

Since its dawn, QM has always posed challenges in its interpretation and compatibility with other theories. These problems originate in the different statuses of an agent (an observer) and a measurement. In other sciences, the agent can collect information about the object without perturbing its properties. Biological experiments typically involve collecting small tissue samples without consequences to an organism or a colony. In psychology, we expose human or animal subjects to forgettable, often fictional, experiences and observe their decisions and reactions. Macroscopic objects and phenomena, such as the atmosphere, oceans, polar caps, rock formations, or celestial bodies, remain unchanged, regardless of how they are observed. It is only at the quantum level that the observer significantly perturbs the system with every observation. Moreover, specifically in QM, measurement outcomes appear to the agent in a random manner, and answers to some questions are restricted to a countable set.

Numerous questions immediately follow from these distinctions of quantum theory. For example, one should wonder if the agent does indeed know everything possible about the system or if there exist some hidden variables supplementing the quantum-mechanical formalism, which would cover the randomness issue, if quantum interactions are relativistically causal or if the agent is described by the same physical principles as the system. If so, then how are they distinguished by being able to conduct a measurement? But if this is not the case, we should ask where the demarcation line between two realms lies. Collections of answers to questions such as these were gathered in various interpretations of quantum mechanics (e.g., Refs. [1,2,3,4,5,6,7,8,9]). Each of these interpretations addresses certain philosophical questions but either ignores or fails to deliver logically consistent answers to some others. In this contribution, we do not attempt to build up an interpretation of quantum mechanics from the philosophical point of view, but we inspect a quantum measurement based on a wider scientific consensus, including electromagnetism and elements of physiology. While such an analysis still leaves some fundamental issues unanswered, this approach would allow us to understand a bit better which features are highlighted by which interpretations and where the transitions are between them.

## 2. Cyanobacterium Evolves to Quantum Physicist

We begin our quest of understanding a quantum measurement by looking at the biological development of sensing. Most generally, a measurement can be understood as obtaining any information about the surroundings. Let us hence inspect the evolutionary development of cognition. Cyanobacteria, the first photosynthetizers on Earth, might have appeared in water about 3.4 billion years ago [10]. This event may mark the first association of physical, rather than chemical, conditions with a survival strategy. The first predators may have appeared as early as 2.7 billion years ago, and the first complex animals evolved ca. 2.1 billion years before today [11]. The relation between the intensity of ambient light and nutrition availability, to both prey and predators, must have affected these early lifeforms. As evolution progressed, more complicated survival strategies based on external conditions had to be developed. For example, a biological cycle had to be adjusted to days rich in solar energy and nights, when resources needed to be spared. Likewise, as plants conquered dry lands about 450 million years ago [12], they had to adjust to harsh winters, for example, by rejecting their photosynthesis organs (leaves) and relying on accumulated supplies, or to the frequency of rain. Naturally, these early adaptations were first achieved by natural selection, rather than conscious choice.

Adapting to external conditions turned out so important that it was beneficial to develop an energy-consuming nervous system about 550 million years ago (see Ref. [13] for the discussion) devoted to reacting to changes in the environment. Eventually, finding patterns in external stimuli became a crucial part of survival strategy, and as such it was linked to the mesocorticolimbic circuit (the reward system), giving rise to scientific curiosity. The significance of these patterns to our existence, a general agreement in their interpretation (from dropping leaves in autumn to using Schrödinger’s equation for predictions about future measurements), and the ability to learn about them from other individuals highlight their external nature and objectivity. Otherwise, why would the agent’s mind generate cryptic information relevant for its continuity and disguise its meaning in imaginary characters? It is rather safe to assume that there is a physical reality outside of us governing our experience and our existence depends on its own laws.

A physical picture of the Universe was, up to the beginning of the 20th century, fairly consistent and comprehensive, based on Newtonian mechanics, electrodynamics, and statistical mechanics. However, a closer inspection of both theoretical predictions and more precise experiments caused cracks in this picture, vividly discussed to this day. On one hand, properties of electromagnetic waves gave rise to special relativity (SR) [14], which reduced the distinction between the space and time dimensions. Later, general relativity (GR) was introduced, defying the absoluteness of spacetime, as it becomes curved in the presence of, now equaled, energy-matter. An example of an unobvious feature of GR is black holes [15], inaccessible regions of spacetime with infinite curvature, now well evidenced [16].

On the other hand, the understanding of light (e.g., the photoelectric effect [17]) and the atomic structure has led to the conclusion that certain physical quantities, such as angular momentum or the energy of electromagnetic radiation, must come in portions. This greatly complicates the description of a system, as it typically involves more than one classically distinguishable state. Such a coexistence, known as superposition, does not have a proper analogue in classical logic; it rather establishes a new relation parameterized by complex numbers. Physically, superposition can be seen as an ability for the system to experience more than one history, provided that they converge at some point. The principle of superposition even extends to complex systems consisting of two or more parts, allowing for entangled states. States from this class exhibit stronger-than-obtainable correlations with local operations and classical communication [18]. In general, the probability of observing a system described by its final state $|f\rangle$, given that its initial state $|i\rangle$ is governed by the Born rule,

(1) $$P\left(|i\rangle \to |f\rangle \right)={\left|\langle i|f\rangle \right|}^{2}.$$

$|i\rangle$ and $|f\rangle$ can be either normalized vectors with some *d* complex components or complex functions integrable to 1 in squares of moduli, and $\langle \cdot |\cdot \rangle$ represents the scalar product. They encapsulate the maximal possible knowledge about the preparation of the system. However, this knowledge is not complete in the sense of a classical phase space, where, say, both the position and the momentum are determined precisely. In QM, different representations of states correspond to eigenbases of noncommuting observables, and vectors from one such basis are necessarily superpositions in another. This results in the Heisenberg uncertainty principle, which proclaims that the product of variances of observables A,B in any state is bounded from below by a quarter of the modulo of the mean value of their commutator,

(2) $$\Delta^{2}\left(A\right)\Delta^{2}\left(B\right)\geq \frac{1}{4}\left|\langle [A,B]\rangle \right|.$$

In a mechanistic fashion, we associate this uncertainty with an unavoidable disturbance of a system due to interaction with the measuring apparatus. Probably the best known example of an explanation of the uncertainty principle is by a transversal illumination. When localizing a material particle on the xy-plane, we would cast a wide beam of photons with sufficiently short wavelength in the *z* direction. The found location would be marked by a shadow on a screen behind the particle. This means that some photons were absorbed and scattered by the particle. The scattered ones exchanged some momentum in the xy-plane with the subject. Physical arguments for other scenarios are often more involved, but uncertainty is a fundamental principle that must play a role.

A more general description of quantum systems, which allows for an incomplete knowledge, is based on mixed states, i.e., normalized, positive semi-definite matrices or functional operators ρ. Mixed states represent statistical mixtures of pure ones and arise naturally in a few contexts. They can correspond to our genuine ignorance about the preparation procedure or appear due to neglecting another system with which ours is correlated. Since systems correlate with one another by interaction, any new formalism of QM is required to include the mixed state description. The measurement outcome *i* of observable *A* is now associated with a projector (a Hermitian operator with eigenvalues 0 or 1) $\Pi_{i,A}$, and the Born rule for the probability of observing value *i* of a given quantity with state ρ is

(3) $$\begin{array}{c} P\left(i|A,\rho \right)=\mathrm{Tr}\rho \Pi_{i,A}. \end{array}$$

With the introduction of QM, SR, and GR, for the very first time, the classical picture of reality accumulated over the history of science has been shaken—from above, in the limit of extremely high speeds, masses, and distances, and from below, by the behavior of the smallest building blocks of reality, in a realm where an act of observation itself influences the experiment. Soon after, it turned out that these two extreme regimes can even be incompatible with each other, with examples of difficulties in formulating fully relativistic QM or quantized gravity theory, the problem of the Universe’s expansion, or the breakdown of quantum formalism in the limit of infinitely many subsystems.

It should come as no surprise that these developments were not immediately accepted by the general public. This was the case especially with quantum theory, even among its founding fathers. Schrödinger proposed a gedankenexperiment with a cat in a sealed box to be driven to a superposition of being alive and dead to ridicule predictions of the new theory [18]. Einstein struggled with accepting the randomness of QM and, together with Podolsky and Rosen [19], postulated supplementing quantum theory with additional parameters intrinsically hidden from observers. This work later stimulated the discussion on hidden variable (HV) models. The conclusion reached up to now seems to be that while theories with general HV models are plausible [2], specific variants, such as local (LHV) [20], a specific kind of nonlocal (NLHV) [21], or non-contextual (NCHV) hidden variable models [22], are falsified by theoretic works supported by accompanying experiments (e.g., Refs. [23,24,25]). Even Freedman and Clauser conducted their pioneering experiments on Bell’s inequalities in 1972 [26] with an expectation that they would actually display obedience to the limits imposed by LHV theories.

## 3. Quantum Physicist Measures System

In quantum mechanics, an agent learns about their surroundings through an act of measurement. The agent interacts with the system in a controllable way to relate to one of its properties and, as they believe, receives a one-time definitive answer. Almost always, such an experiment must be repeated a number of times to establish some statistics. An act of measurement is associated with either the destruction of the system or an update on future predictions concerning it. Once the quantum mechanical formalism of state vectors and observables is accepted, certain pairs of measurements are incompatible by the Heisenberg uncertainty principle. They cannot be faithfully conducted simultaneously or in sequence, as one of them disturbs the outcome of the other.

These features of QM called for an interpretation that would bring it closer to the legacy of empirical sciences. Such an interpretation should address questions such as “what is a measurement?” (which is one possible formulation of the big measurement problem according to Pitovski [27]), “who or what is the agent and what is their role in the act of measurement?”, “why does the agent report only one outcome of the measurement?” (Pitovski’s small measurement problem, ibid.), or “what can we deduce about the underlying structure?”. As QM was the first inevitably random theory, a particularly interesting matter for these interpretations was “is there a reason that this particular result and not any other was reported in a measurement?”, effectively reviving the discussion on possible HVs. Let us briefly review some of the most highlighted examples of interpretations of quantum mechanics and explore their different takes on the measurement problem.

*The ensemble interpretation* has been promoted since Born’s 1926 work [28] on quantum scattering by himself and Einstein. Undeniably, as QM only predicts the probabilities of certain events, it seems natural to say that it only applies to large collections of systems, rather than to individual instances. In this proposition, QM is not applicable to individual systems, which should not even be considered. As individual events are not a part of this approach, it is effectively impossible to properly pose the measurement problem here.

We also have *the Copenhagen interpretation*, in which an observer conducts a measurement of their choice. The system responds by a reduction in its initial state and yielding an output through the measuring device so that the retrieved outcome corresponds to the state the studied system was driven to by the measuring device. There is a clear distinction between the classical macro-reality of the agent and the quantum micro-reality of the system. An important part of the Copenhagen interpretation is the *collapse postulate*, which proposes that the state of a system is updated to its projection onto a subspace corresponding to the observed outcome. Thus, a measuring apparatus induces a sudden, stochastic jump in the evolution of the system. While the distinction of micro- and macroscopic phenomena is not investigated in this school, the physical principles of the measuring device could certainly be explored further.

The *de Broglie–Bohm theory* [1,2] was based on a reinterpretation of the Schrödinger equation as a system of Hamilton–Jacobi equations. The distribution of the position of a particle is described by the squared modulo of a wave-function, while momenta are given by a certain field defining the complex phase of the wave-function, corresponding to each location. Having these two quantities, we can define a precise trajectory for every initial location, which we cannot know with infinite precision. We thus recover classical dynamics at the expense of introducing a quantum potential, the nature of which is nonlocal.

The collapse postulate apparently violates the time-reversal symmetry that we expect from physical theories. A motivation for *time-symmetric interpretations*, which exploit the *two-state vector formalism* [4], was to restore this symmetry. As a solution, a formalism was introduced in which predictions are made with forward- and backward-evolving wave-functions: one traced from a source, the other from a detector. Such an interpretation claims that the relevant effects appear only at the overlapping fragments of the two states. Predictions based on the two-state vector formalism fail to reproduce the Born rule. The transactional interpretation [6], with the main difference being that both sources and detectors produce the advanced and the retarded wave-function, is claimed to have improved in some respects, but it has not been discussed in light of the newest arguments. Another objection is that certain experiments, e.g., those based on entanglement swapping, can have a complicated, or even undefined, causal structure.

*Everett’s* interpretation [3] states that at the (rather loosely defined) moment of measurement, the state of the Universe splits to many branches, each corresponding to a different outcome. It was cursed by being dubbed the “many worlds” interpretation for the purpose of popularization. In this version, an act of measurement summons new physical copies of the reality, each realizing consequences of a different outcome. It was never a suggestion by Everett that upon a measurement the physical substance of the Universe multiplies. Instead, new components of some global wave-function appear. The superposition, though fundamentally different, is closer to the logical OR operation, rather than AND or the Cartesian product.

In the class of *objective collapse* theories, the Ghirardi–Rimini–Weber (GRW) model [5] sees the Schrödinger(-type) equation,

(4) $$i\hbar \frac{\partial |\psi \rangle }{\partial t}=H|\psi \rangle$$

(where Hamiltonian *H* can be of the Dirac type), as an approximation of the Itô equation, the nonlinear terms of which bring it closer to the Lindblad–Kossakowski–Rossini–Sudashan evolution of mixed states [29,30]. Physically, we can interpret these nonlinearities as random convolutions of the ontic wave-function (describing real properties, not knowledge) with Gaussian filters, describing Brownian-like motion. Diosi was the first to attribute these reductions to observable differences in spacetime geometry [31], but given that the gravitational force is extremely weak and the frequencies of gravitational waves observed in the collisions of black holes and neutron stars are of the order of 30 Hz, we can expect very large coherence lengths of typical gravitons. Moreover, the GRW model violates the energy conservation law. Due to experiencing kicks, in these models a system may gain kinetic energy linearly in time. However slow this gain may be, it can build up to significant quantities.

*The relational interpretation* due to Rovelli [7] rejects the idea of an objective state of the Universe. Rovelli adopts the idea of the agent becoming entangled with the object of the measurement. In this interpretation, all entities are treated on equal footing as quantum objects. While for one observer a system already has a definite property, the other perceives the state of the system and the first observer is in an entangled state until they perform their own measurement. In essence, this point of view is based on the Wigner’s friend scenario.

*QBism* [9], or quantum Bayesianism, is a radical minimalistic philosophical point of view, which only acknowledges the agent and their experiences. As these experiences accumulate, the agent updates their predictions about future events, eventually reaching the Born rule. This is the only interpretation that denies any underlying physics, including the free evolution of the system. In this respect, it is the closest to the surrendering “shut up and calculate” approach, in which we accept our formalism simply as machinery for making predictions.

Considering a plethora of interpretations and their opposing propositions about measurements, various positions can be taken. Those primarily interested in an operational approach tend to adopt the Copenhagen interpretation. Others who are more concerned about coherence of the underlying picture will probably opt for the Everettian interpretation but will face the criticism of many worlds. Yet another group, discouraged by the fact that none of the existing interpretations is free of problems, or simply not needing to ask any further, will likely settle for QBism. Those attached to concepts of classical physics might favor the de Broglie–Bohm picture, but the accompanying formalism is not much exploited.

Many interpretations treat the act of measurement as a given primitive. Mostly its consequences are considered, but not its internal structure. Even if the measuring device and the agent are treated as quantum-mechanical objects, this is performed by simply assigning them a quantum state, without much attention paid to the underlying structure and the environmental context. In this contribution, we propose decomposing a quantum measurement to more basic processes. Each of them should be recognizable in a generic description in possibly many measurement techniques and should be followed by clear physical intuition.

Likely the first attempt of a deeper analysis of a quantum measurement is due to von Neumann, who imagined that it is conducted by letting the system interact with some measuring device *M*. *M* is as much at the quantum level as the system, initialized in a known state, and in von Neumann’s model, after the interaction, both the system and the measuring probe are in a single, definite state corresponding to one of the possible outcomes. Finally, the agent extracts the information from the measuring probe, leaving the system untouched.

A more detailed description than the one presented here may differ depending on the type of system or the measured quantity and the measuring apparatus, but one possible framework is described below. The motivation is provided by three partially overlapping philosophical assumptions: *physicality*, meaning that our accumulated experience reflects the underlying physical reality, which relies on billions of years of the legacy of sensing in living organisms, *frugality* (William of Occam’s razor principle), postulating that every new notion must be well motivated and related to accepted physics, and, finally, *antianthropocentrism* (the Copernican principle), requiring that physical laws are not subject to the presence or participation of an agent. On one hand, the last principle is motivated by our successes in describing the dynamics of quantum systems with the Schrödinger equation; on the other, it relieves us from discussing what constitutes the agent.

A quantum measurement is an intentional arrangement of the evolution of a system and its environment, such that it leads to a correlation between the measured property of the system and the internal state (representing their knowledge) of the agent. Note that decoherence (the inability to track the full quantum evolution any further) is a broader notion than a measurement. A system could couple to orthogonal states with identical photon statistics (states differing only by phases in a standard photon number basis), the difference between which could not be directly accessible to the agent. It is also not enough to require spectrum broadcasting (a form of broadcasting, in which state every sufficiently large fragment has the same spectrum as the state of the system [8]), but rather that many consistent signatures of a given outcome are broadcast.

A measurement will involve a few entities. First, an important part is the agents themselves, who conduct it. We also need to distinguish some quantum system we want to study and some instrument to perform it with. The agent and the device will have some richer inner structure. We shall also assume some medium, which is a system (a field) coupled both to the measuring apparatus and the agent. Finally, we will use the concept of an unspecified environment, a part of the global quantum realm, the details of which the agent will remain ignorant of.

The first stage is *transformation*. Because we are only able to register events well localized in spacetime, most often the agent needs to couple the quantity they want to study, the position or times of arrival, to the measurement device. In technical terms, at this stage, we map the desired pointer basis (states distinguishing the values of studied properties) to a sub-basis of localized states. In optics, this could be realized by a system of mirrors and phase plates; for spin measurements, it is rather an arrangement of magnets. Technically, these transformations are nearly perfect realizations of the unitaries of the system, and therefore they should be considered as reversible. As measurements are not necessarily compatible with one another, this is the stage where the agent might exercise their free will (if there is such a thing) by arranging one setup and not another.

We can then distinguish *conversion*, during which the system interacts with a detector. The role of the detector, as understood here, is to provide an interface between the system and the medium, which can pass some signal to the agent. A textbook example is the moment when a photon hits the first cathode in a photomultiplier. It is consequently replaced by a photocurrent, which can be conveniently amplified to macroscopic levels. A more modern case is that of transition edge detectors, where the system excites a phonon in a superconducting quantum interference device (SQuID), resulting in a change in the magnetic flux. Typically, the system–detector interaction is very weak, giving a minuscule probability of reaction (understood in a classical way). To increase this probability, but also for manufacturing convenience, an experiment would rather involve an array of such elementary detectors.

Immediately after the conversion, *amplification and broadcasting* starts. The amplification does not need to be in terms of the energy of the signal, though most often energies associated with the system are too low for the agent to register. More importantly, it is the amplification of coherence, transforming a superposition into a multipartite entangled state. If a superposition of states of the system was an applicable description up to this point, now it refers to states also describing the medium by which the agent will be communicated with, e.g., light, and other factors. For example, optoelectronic sensors include actual amplifiers (often transistor-based) for photoelectrons. A grain on a photosensitive layer absorbs or scatters photons falling onto it. It was pointed out, e.g., by Żukowski and Markiewicz [32], that this amplification does not need to couple the orthogonal states of the system–detector with the orthogonal states of the medium. Consider the following interaction between a system *S* and a medium element *M*:

(5) $$\begin{array}{c} {|0\rangle }_{S}{|0\rangle }_{M}\to {|0\rangle }_{S}{|0\rangle }_{M},\\ {|1\rangle }_{S}{|0\rangle }_{M}\to {|1\rangle }_{S}\left(\cos\alpha {|0\rangle }_{M}+\sin\alpha {|1\rangle }_{M}\right). \end{array}$$

Setting α=π/3, we obtain that the probability of confusing the two outcomes based on the medium Pcos(π/3). Consider now coupling to *N* quanta of the medium:

(6) $$\begin{array}{c} {|0\rangle }_{S}{|0\rangle }_{M}^{⊗N}\to {|0\rangle }_{S}{|0\rangle }_{M}^{⊗N},\\ {|1\rangle }_{S}{|0\rangle }_{M}^{⊗N}\to {|1\rangle }_{S}{\left(\cos\alpha {|0\rangle }_{M}+\sin\alpha {|1\rangle }_{M}\right)}^{⊗N}. \end{array}$$

Taking the example of N=10 and α=π/3, we arrive at the probability of confusing the two outcomes:

(7) $$P\left(0↔1\right)=\cos{\left(\pi /3\right)}^{20}\approx 9.5\times 10^{-7}.$$

Thus, the distinguishability between two outcomes that may arise from repetitions is a weakly entangling interaction. For example, a few scattered photons may give poor and noninformative statistics, but a stronger illumination allows us to resolve a position of, e.g., a gauge’s hand.

It shall be pointed out that in practice the medium almost always involves the electromagnetic field. An unusual example can be that of a Geiger–Müller counter, where a detection is primarily announced to the agent by an audible click, but it originates in electric discharge in a gas. Electromagnetic radiation is omnidirectional in natural situations, and it propagates with the speed of light, making it particularly difficult to control. Not only is there an excess of information about the system’s state broadcast, but also its future evolution remains unknown. Involving the electromagnetic field itself greatly contributes to the effective impossibility of a precise theoretical description of a quantum measurement.

The final stage of the measurement process is *perception*. It starts at the moment that information in the medium reaches the agent to stimulate their senses. In this way, they become coupled to the system and are ready to report an outcome. On the other hand, perception lasts as long as the agent contemplates their correlation with the system in this particular act of measurement. The agent’s own reaction to the outcome contributes to the amplification described above. By the time the agent realizes the outcome, the information about the system’s property is already incoherent (classical). The role of this stage is to implement the collapse of the state, i.e., constrain the description of the system relative to the signal received from the environment.

von Neumann’s approach resembles the one depicted above. One similarity appears with the introduction of the detector, here called *M*. However, a lot is left unexplained. For one, the nature of the interaction between the system and the measuring probe is not discussed. In fact, it was later shown that within unitary evolution it is impossible to drive from a general state to the one, which *M* reflects as a unique value of the property of the system [33,34]. Note that von Neumann aimed at justifying, rather than eliminating, the wave-function collapse, as the Copenhagen interpretation was the most mature one at the time of coining this understanding. Thus, the collapse must be included in this interaction. The other missing description is the extraction of information from *M*. Since, however, it is already classical and deterministic, one can easily imagine the physics of this process.

Another analytical approach to the measurement problem was attempted, e.g., by Auffèves and Grangier [35]. One difference between their and the current work is that Ref. [35] features the modality–system–context approach, where the context is what we described as a transformation and modalities are states associated with measured quantities. That approach is aimed at a deeper abstraction, rather than a physical picture of the measurement.

One additional method that deserves mention here is intrinsic measurement. It is a procedure in which we let the system evolve further if it has a desired property and we dump or destroy it otherwise. An example is a polarizing filter of light. It is a measurement in the sense that we conclude the value of the property from further consequences. Still, the essence of this procedure is compatible with the framework presented above, as in conclusion we perform a usual measurement on the surviving copies.

## 4. A Quantum Physicist Wants to Have a Cake and Eat a Cake

Let now us consider the feasibility of reversing a measurement. This is a key ingredient of the considerations of the Schrödinger’s cat [18] and Wigner’s friend [36] gedankenexperiments, which, in turn, serve as a motivation for some interpretations (e.g., relational). One argument is to consider the complexity of the classical representation of the state of the systems involved. Modern everyday computers allow us to handle arbitrary mixed states of more than a dozen qubits (two-dimensional quantum systems). Supercomputers could deal directly with a few dozen qubits. That is to say, these machines can hold such generic states in memory and perform some simple operations on them, e.g., calculate the mean value of some operators. The main obstacle in attempting a more general modeling is that multiplying two *d*-dimensional matrices requires $O(d^{3})$ floating point operations. Clearly, these numbers are quickly saturated in the development of a measurement.

The computational and operational complexity of the problem will always be an argument that the reversibility of quantum measurements is not attainable *in practice* rather than *in principle*. As it seems, however, the analysis cost escalates so rapidly beyond any practical feasibility that it is safe to say that the reversal of a measurement is impossible *in principle*.

At this point, one may blame the measuring device and the medium for decoherence. A tempting solution might be to eliminate these elements and to feed a quantum system directly to the agent’s eyes in hopes that a superposition will somehow reach their mind. The minimal light intensity that can cause a reaction in the human brain is estimated to be between five and nine visible photons within a time window of 100ms per rod cell. Producing entangled states of that many photons is well within the reach of modern quantum optical experiments [37]. However, once the state reaches the agent’s eyes, all entanglement is immediately lost due to the extremely convoluted mechanics of biological systems. Finally, at the stage of perception, the medium acts on the agent’s organs to produce an impression. A neuron transmits electrical signals not by electrical conductance but by generating membrane potentials. In simple terms, stretching and contracting a membrane opens and closes exchange channels for sodium and potassium cations, which differ in size. Given that the physiological saline has NaCl condensation of about 1% and neurons have lengths of about $10^{-5}$ m, potentially hundreds of cations could be involved in transmitting an individual nerve pulse by a single nerve cell. Information becomes classical immediately upon reaching the agent, at the very latest. This leaves no space for any practical realization of the Schrödinger’s cat or Wigner’s friend experiments. Just like in the case of the Universe, the unitary description of Wigner’s friend(s) may be still formally correct, but it is not feasible and therefore not relevant anymore.

A consequence of the escalating complexity of a quantum system is decoherence. However, it refers to the lack of knowledge of the agent and not an erasure of information per se, which is often reflected in the phrase “Nature would know”. While for the measurement we required that a large number of consistent signals be broadcast, to maintain coherence we need to keep track of every single system that could be involved in the measurement process. An observer may need some significant Hamming distance between states representing different outcomes, but we can speculate about a “superobserver”, who would have an infinite determinacy to resolve the measurement by amplifying any differences in the state of the environment. The agent attempting to reverse measurement is destined to fail if they miss or wrongly describe a single entity potentially involved.

The central concept of both the Schrödinger’s cat and the Wigner’s friend gedankenexperiments is the sealed box, in which all the quantum evolution of the encapsulated system is contained. However, the interior of such a box would need to be perfectly reflective of the whole electromagnetic spectrum, which is not possible. The box exchanges heat, and therefore information, both with the interior and the exterior. So, Wigner would need to keep track of and control the exact state of his friend at the atomic level, the whole electromagnetic field inside the box, the thermal oscillations of the constituents of the box, and its radiation.

For the sake of argument, we could even grant Wigner the box reflecting all the photons inside it, such that the system inside can remains coherent. Then, the problem is that he still needs at some instance of time to know or control all microscopical parameters of the interior and exchange this information in an extremely confined way, so that no information leaks out of the Wigner–box interface; otherwise, coherence is lost.

In his own manifesto about the quantum measurement problem, Brukner [38] writes:

While on the one hand, measurements have to result in irreversible facts (otherwise, the notion of measurement itself would become meaningless, as no measurement would ever be conclusive), this irreversibility on the other hand must be merely FAPP if quantum theory is in principle applicable to any system. Any system means that the measuring apparatus itself can also be subject to the laws of quantum theory. My main point is the following. While it is obviously possible to describe the subject as an object, it then has to be the object for another subject. In my eyes, not enough thought has gone into the fundamental nature of FAPP. More research on the philosophy of FAPP, if you like, should be done by philosophers of physics.

Here, the acronym “FAPP” stands for “for all practical purposes”, in contrast to “fundamentally”, and is due to John S. Bell. The main message of the current work is not contradictory to this quote but reverses the emphasis. The message to be conveyed here is that before we can consider any departure from the laws of quantum mechanics, Schrödinger’s Equation (4) becomes impractical, similarly to Bernoulli’s abandonment of the kinetic description of gases in favor of statistical treatment.

If a dramatic escalation of the description’s complexity is not a sufficient argument against the full control of the argument against the feasibility of quantum measurement reversal, causality should be. Practically any measurement procedure involves an electromagnetic field. Hence, amplification is dependent on events from beyond the agent’s light cone, e.g., some sudden moves of atoms interacting with radiation.

Consider the example of the Bell experiment with quasar light used to drive the polarizers defining the measurement settings [39]. Every building block of this realization, the production of light in nuclear fusion, propagation through space, coupling to an electronic system, parametric down-conversion, etc., can be in some fashion described quantum-mechanically, but such a statement is at a different level than claiming that photons created billions of light years apart in stars drove an experimental setup in a different region of the Universe to show a violation of Bell’s inequality.

## 5. The System Does and Does Not Obtain a Description

In the remaining, we shall discuss the basic issues related to the measurement problem. In the center of the discussion is the question of the ontic or epistemic nature of the wave-function, but we will also address other formulations of the measurement problem.

The first matter on the agenda is recognizing the actual moment in which the measurement happens. From the point of view of the Copenhagen interpretation, we can define this moment as the one in which we depart from the Schrödinger equation. In the orthodox interpretation, this happens at the collapse. When looking into the physics of the measurement, this would rather be a time at which are unable to apply Equation (4) anymore due to the complexity of the system. One could debate if the Schrödinger equation can be in principle used to describe the entire Universe, but such an attempt will only be illusionary. In practice, this or any other equation can be useful only for its very small fragments.

By definition, transformation is a unitary operation on the system, which means that it can be undone by using the same device. Naively, we would believe that conversion by the detector can also be seen as coupling two relatively simple systems, but typically “elementary detectors” appear in a large number—to increase the collection rate and due to the convenience of manufacturing. Thus, already at the stage of conversion, keeping a record of the evolution may become extremely difficult.

In contrast to transformation, amplification is specifically defined as correlating the system (through a detector) with as many particles in the medium as possible. Note that the medium is not designed to focus directly on the agent, but it rather spreads across the whole space. The agent only receives a small part of the medium, and the vast majority of it propagates in a void or to other agents, leading to the inter-subjectivity of observations.

As we have discussed, perception is an extremely complex process that happens inside the agents themselves. Even if quantum information reaches their senses, it becomes immediately classical.

A similar approach in which the measurement occurs due to our inability to provide a description was initialized already in Ref. [40]. However, Zeh primarily focused on chaotic evolution within the measurement device. He believed that the main mechanism for decoherence was phase randomization. Much of the work on the role of decoherence in the measurement process was conducted by Żurek, who proposed a mechanism of quantum Darwinism [8].

The picture presented above does not mention any physical collapse mechanism. Indeed, it would be unphysical, at least in terms of locality. The report of a certain outcome by the agent immediately implies the confirmation of the same result by all other potential observers, including those still outside of the light cone of the first one. One possible resolution is to basically accept the superluminality of a collapse. Indeed, a single outcome of a quantum measurement cannot be used to communicate a message, as we perceive it as random, but still, this exception may be troublesome.

Another possibility would be to allow different perceptions between the observers. Actual quantum experimentalists do not report such disagreements, and the objectivity of quantum measurement results lie at the heart of important protocols such as teleportation [41] or quantum cryptographic key generation [42]. It would be extremely conspirative to believe that the state of the agent is such that the measurement would drive them to the delusion of a different outcome. Thus, the detailed state of the agent is irrelevant.

There is also a third way out, which is to assume that formally the agent is still in a superposition with the system, the detector, and the medium. Each branch of the medium state contains consistent information about detection that any number of agents can receive. In each branch, every agent has an impression of a unique outcome, and they all agree about the outcome. However, since it is impossible to accomplish any transformation between branches anymore, it is most reasonable not to consider other possibilities, giving rise to a collapse, at least in the mental state. The same logic would then apply to implicit measurements.

While not in a direct relation to the measurement problem, an important argument is the discussion of entanglement [18] and the Einstein–Podolsky–Rosen correlations [19], which by the Bell theorem [20] lead to a refutation of statistical models with local hidden variables. These topics recently gathered popular attention due to frequent reports on progress in quantum technologies, acknowledged by a Nobel prize in physics for the year 2022.

For this consideration, we will not include the state of the environment after the broadcast. As shown above, it suffices to assume that the environment copies the information in the pointer basis for each component of the wave-function.

Consider a singlet state of two-qubits,

(8) $$|\Psi_{-}\rangle =\frac{1}{\sqrt{2}}\left({|0\rangle }_{A}{|1\rangle }_{B}-{|1\rangle }_{A}{|0\rangle }_{B}\right),$$

where $\sigma_{z}|i\rangle ={\left(-1\right)}^{i}|i\rangle \;\left(i=0,1\right)$ and A,B are two arbitrarily separated systems. When $\sigma_{z}$ is measured at both sides, the state predicts that any of the agents observe (that is to say in the collapse-less scenario, they correlate with the amplified states of subsystems) the outcome “+1”, while the other would observe “−1”.

Now, let us discuss the case in which the pointer basis (i.e., the basis in which coupling with the detector is block-diagonal with respect to the system) is $\left\{|0\rangle ,|1\rangle \right\}$ on one side and $\left\{|\pm \rangle =\left(|0\rangle \pm |1\rangle \right)/\sqrt{2}\right\}$ on the other. Then, the Born rule predicts an equal probability for all four possible outcome combinations.

Finally, let both agents measure $\sigma_{x}=|+\rangle \langle +|-|-\rangle \langle -|$. As $|\Psi_{-}\rangle$ is invariant under any pair of identical transformations at each side, the predicted statistics are the same. Specifically,

(9) $$\begin{array}{ccc} & & |\Psi_{-}\rangle \\ = & & \frac{1}{\sqrt{8}}\left(\left({|+\rangle }_{A}+{|-\rangle }_{A}\right)\left({|+\rangle }_{B}-{|-\rangle }_{B}\right)\right.\\ - & & \left.\left({|+\rangle }_{A}-{|-\rangle }_{A}\right)\left({|+\rangle }_{B}+{|-\rangle }_{B}\right)\right)\\ = & & \frac{1}{\sqrt{2}}\left({|-\rangle }_{A}{|+\rangle }_{B}-{|+\rangle }_{A}{|-\rangle }_{B}\right). \end{array}$$

As can be seen from Equation (9), each combination of outcomes perceived by the agents is described by two histories (two terms in the middle of this equation), which are coherent with one another.

In interpretations with a wave-function collapse, this directly refutes local realistic theories, in which the results of local measurement are predefined regardless of experimental arrangements in other locations [20].

When a collapse is accepted, the Bell theorem has three assumptions. The first is *realism*, referring to the Einstein–Podolsky–Rosen *elements of reality* properties of the system, which can be predicted with certainty without an additional disturbance. In each run of the experiment, the outcomes should be predefined for all possible local choices of measurements. The second is *locality*, responsible for agreement with causality. It states that the choices of observables by one observer cannot instantaneously influence events in a distant laboratory of the other. Finally, there is a strong assumption of *free will*, stating that observers are free to choose their measurement settings independently of past events. With the second assumption motivated by SR and the third being impossible to debate on, if one of these assumptions needs to be challenged, it is most commonly realism.

Consider now the point of view where we do not take the collapse of a wave-function as physical and we accept that the agent cannot actively change the amplitudes. The assumption of *realism* does not make sense, because all possible outcomes occur in different timelines. The *free will* assumption is still unverifiable, but this time we lean towards our choices being determined. Consequently, the second assumption is ill defined, as the events in two different locations have a common cause. The Bell theorem question thus reduces to a question of how superpositions govern evolution, even at long distances. Without true means to falsify this conjecture, we could be forced to accept this fact in a QBistic fashion.

A Copenhagenist or a QBist would welcome an acknowledgment of the distinguished role of the agent. It is currently, and perhaps always will be, unfeasible to combine quantum mechanics with what we consider free will or consciousness, so we rather postulate these attributes. Moreover, it is in the spirit of these two interpretations that a measurement would involve the will of the agent. With the lack of understanding of free will, the current status does not allow us to judge about the quantum or non-quantum nature of the agents themselves.

Nodding to the Everett’s interpretation, we observe that our model of measurement does not include any model for a physical collapse. The agent experiences different measurement outcomes in different timelines, which may as well continue to infinity. In each timeline, there is a consistent historical record leading to the present moment. However, as there is no further connection between them due to an extremely complex evolution, we experience only one timeline, and others are only hypothetical.

What about the state? Is it a property belonging to a quantum system or a mere mathematical tool? The Everett and, to some degree, Copenhagen interpretations stress its physical relevance to the system. However, consider an indestructible quantum entity, for example, the spin of a particle. At some point in time, we describe it with a pure state, but we could only draw such a statement from a measurement. A Copenhagenist then refers to a collapse, but as we discussed, there is no physically motivated model for it. In Everett’s and QBistic interpretations, rejecting a hypothesis that our spin never interacted with any other part of the Universe, we have to assume that the state is a Bayesian concept referring to our knowledge, updated only at some recent time with our experience. Therefore, it does not describe any correlations that spin could have acquired earlier.

Also, we can ascribe states to not-yet-existing systems based solely on the experience from past runs of experiments, such as photons from a pulsed source. We are able to predict the behavior of not-yet-emitted particles not by applying the Schrödinger equation to the entire system but solely based on previous experience. As QBists stress, it suffices that we master the betting strategy for measurement outcomes.

Finally, one could ask about the underlying physical reality. QBism refuses to discuss any physical realization focusing specifically on the experience of the agent. This approach seems to be convergent with the point of view of, e.g., Zeilinger, who occasionally expresses his opinion that “photons are just clicks in detectors” [43]. Indeed, it is impossible to infer the nature of the underlying reality based on experience. However, taking this observation as an imperative seems very radical, as it defies the essence of science, which is to find relations between our observations in order to make some predictions about future events. These relations require a language to describe them. The language of mathematics provides necessary technicalities but lacks the essence. A way of describing phenomena as a series of collisions of particles proved itself to be exceptionally versatile and useful, even if not complete. Thus, particles exist at least at the same level as numbers—as concepts. The rules dictating which collisions are more effective than others (conservation principles, selection, and superselection rules) may be treated as fundamental or explored in a separate theory. Likewise, we should accept the evolution of these particles as given. Contrary to QBism’s denial, physical laws should apply regardless of the role of the agent. On the other hand, an inspection of a measurement process does not lead to the Born rule, yet it is, in a QBist’s words, the best betting strategy. We cannot know what the physical reality is, only how it works.

One of the discussions on this problem is due to Leggett [44], who considered three possibilities concerning the physicality of a quantum state. The options are that QM is a complete description and describes a reality, that it does not describe a reality but cannot be supplemented by any other elements, or that it is incomplete. Leggett reached the conclusion that the second option is, by far, the most plausible. The core of his argument is that the superposition of Schrödinger’s cat does not correspond to any observable phenomenon. On the other hand, according to Ref. [44], objective, extra-quantum-mechanical decoherence mechanisms would hinder the realization of the superposition of larger objects [45]. Let us point out that Leggett’s conclusion does not depart from our assumption of physicality but rather is its celebration. Rejecting the other options, he referred to our observational experience. Also, Leggett does not escape the trap of believing in assigning a state vector to a cat, whereas living forms are by definition correlated with the environment.

## 6. Conclusions

The primary goal of this essay was to put the measurement problem in a wider context and confront it with chosen interpretations of quantum mechanics. First, we summarized the evolutionary development of cognition to emphasize the physicality of our observations. We then provided a short overview of chosen interpretations of quantum mechanics to demonstrate the philosophical spectrum of approaches to the measurement problem.

After that, we discussed a decomposition into four stages: transformation, conversion, amplification and broadcast, and perception. Each of these stages was discussed physically, leading to our main conclusions.

Except for transformation, all other stages dramatically increased the complexity of the account of the process. This leads to the unavoidable inability to describe the whole process by means of the Schrödinger equation and, consequently, decoherence. The presented picture suggests that the agent concludes about the measurement outcomes by receiving a small fraction of consistent signatures of the coupling to the system’s respective state, letting the agent update their description. Other branches of the global wave-function have multiple signatures of other outcomes, so they never interfere with one another and become irrelevant.

This description emphasizes certain aspects of the most popular interpretation. Like in Everett’s proposition, the wave-function constantly branches to consequences of all possible events. Much like in the Copenhagen interpretation, an update of a wave-function occurs, but only as the agent’s acknowledgment of the registered events. Similarly to QBism, the wave-function and the Born rule are only tools to compute the probabilities of the agent reporting a specific outcome.

Different interpretations of quantum mechanics do not originate in disagreements but in emphasizing various aspects of the same phenomena. Therefore, we should not spare efforts in their unification.

## Data Availability

Data are contained within the article.
